# Identification of lumbar disc disease hallmarks: a large cross-sectional study

**DOI:** 10.1186/s40064-016-3662-7

**Published:** 2016-11-14

**Authors:** Jun Zhang, Fei Zhao, Feng-Liang Wang, Yong-Feng Yang, Chen Zhang, Yue Cao, You-Lin Wang, Xiao-Juan Shi, Yi Wan, Min Zhang, Meng-Qiao Liu, Chun-Guang Zuo, Hai-Qiang Wang

**Affiliations:** 1 Department of Orthopaedics, Baoji Municipal Central Hospital, Baoji, 721008 Shaanxi Province People’s Republic of China; 2 Department of Orthopaedics, Xijing Hospital, Fourth Military Medical University, 127 Changle Western Road, Xi’an, 710032 People’s Republic of China; 3 Department of Orthopaedics, The Third Hospital of PLA, Baoji, 721004 Shaanxi Province People’s Republic of China; 4 Brigade of Undergraduates, Fourth Military Medical University, Xi’an, 710032 People’s Republic of China; 5 Department of Health Service, School of Public Health, Fourth Military Medical University, Xi’an, 710032 People’s Republic of China

## Abstract

**Background:**

Lumbar disc disease has a disabling impact on global people with heavy burden on society, mainly consisting of lumbar disc degeneration (LDD) and lumbar disc herniation (LDH). The recently released lumbar disc nomenclature version 2.0 deepens our understandings on the diseases. Consequently, there is an urgent need to clarify the occurrence and distribution features of LDD and LDH in a large-scale sample in terms of the novel version.

**Question/purposes:**

We asked: (1) Is there a difference in the occurrence and distribution hallmarks of LDD and LDH in a population-based large-scale sample? (2) Does the novel nomenclature version bring novel vision on lumbar disc disease?

**Methods:**

Five thousand two hundred eighty-eight consecutive cases (26,440 lumbar discs) undergoing lumbar spine MRI were retrospectively included from Jan 2008 to Dec 2010 in a territory university hospital. Five hundred nine cases were excluded. There were 2727 males (51.57%) and 2561 females (48.43%) with a mean age of 43.73 years. Both T1 and T2 weighted lumbar MRI images from L1/2 to L5/S1 were profoundly analyzed in axial and sagittal planes. We classified lumbar discs in terms of version 2.0.

**Results:**

The occurrence of LDH and LDD was 14.18 and 44.23% in average, respectively. Notably, lumbar spine discs were more prone to LDD than LDH. L4/5 was the most frequent level in terms of LDH (26.08%) and LDD (56.09%), followed by L5/S1 (LDH: 24.09%; LDD: 55.33%), then L3/4, L2/3 and L1/2 in ranking order. The prevalence of LDH and LDD in upper lumbar discs from L1/2 to L3/4 was significant lower than the average prevalence rate (*P* < 0.05). The mean age was 24.70 (±14.81) years for normal lumbar discs; 49.76 (±14.95) years for LDD; 37.01 (±12.91) years for LDH; 51.31(±15.00) years for LDD and LDH (*P* < 0.05). Modic changes, HIZ, spondylosis deformans and decreased disc height were linked with older age; whereas Schmorl node and lumbar disc sequestration were not associated with age (*P* < 0.05).

**Conclusions:**

The prevalence of LDD is 44.23%, higher than LDH as 14.18%. L4/5 and L5/S1 are the most frequent involved segments for the majority of lumbar disc diseases. Schmorl node occurs (1.6%) more frequently in upper lumbar spine, independent of age. Modic changes (0.87%) are closely related with older age.

**Clinical relevance:**

When diagnosing and treating lumbar disc disease, it might be important to consider the updated nomenclature of LDD and LDH. Our study provides additional novel vision on the features of LDD and LDH in a large-scale sample based on the nomenclature of novel version.

## Background

In general, lumbar disc disease comprises both intervertebral disc degeneration (MeSH Unique ID: D055959, introduced in 2012) and intervertebral disc displacement (MeSH Unique ID: D007405, corresponding to lumbar disc herniation commonly noted). As the main contributor to low back pain and sciatica, the disease greatly affects people’s work, daily lives and quality of life (Balaji et al. 2014; Lagerback et al. 2015; Thaler et al. 2015), even permanent neurologic deficit and lifelong incontinence due to cauda equina syndrome (Todd 2015). Notably, over 380 thousands patients in USA had to undergo surgical treatments due to lumbar disc disease during between 2000 and 2009 (Yoshihara and Yoneoka 2015), which is only the tip of the iceberg. The majority of patients with lumbar disc disease seek for conservative treatment. In 2013, more than 1 million patients received an epiduralglucocorticoid injection in USA (Racoosin et al. 2015), let alone those outside USA and those seeking for other treatment methods within USA.

Despite its disabling impact on global people at all ages, the occurrence and distribution features of lumbar disc disease remains largely undefined. Notwithstanding novel diagnostic imaging methods have been identified (Arpinar et al. 2015; Lagerback et al. 2015), the gold standard for grading lumbar disc degeneration (LDD) is still based on T2 weighted MRI of the lumbar spine proposed in 2001 (Pfirrmann et al. 2001). In the classic study, Pfirrmann et al. proposed the 5-grades algorithm following evaluating 300 intervertebral discs (IVDs) in 60 patients. So far, the cross-sectional studies with the largest sample size were less than 2600 cases, addressing the hallmarks of lumbar disc disease using MRI (Samartzis et al. 2011; Teraguchi et al. 2014, 2015).

In 2014, the combined task forces of the North American Spine Society, the American Society of Spine Radiology and the American Society of Neuroradiology released the state-of-the-art lumbar disc nomenclature: version 2.0 (Fardon et al. 2014a, b). The updated terminology on LDD and lumbar disc herniation (LDH) greatly expands our understandings of LDD and LDH, starting a new era for scientific community and patients. We asked: (1) Is there a difference in the occurrence and distribution hallmarks of LDD and LDH in a population-based large-scale sample? (2) Does the novel nomenclature version bring novel vision on lumbar disc disease?

Therefore, it is of vital importance to revisit the issues of LDD and LDH in terms of the novel lumbar disc nomenclature. Bearing this in mind, we aimed for achieving the goal using a large cross-sectional image samples.

## Patients and methods

### Study population

Following institutional review board approval, a hospital-based image study was initiated to evaluate the epidemiologic phenotypes of LDD and LDH. First, All lumbar spine MRI images were extensively reviewed from Jan 2008 to Dec 2010, including those patients prescribed by both out-patient and in-patients clinicians due to various factors. Second, the cases with infections, neoplasms, deformities and congenital anomaly were excluded. Third, degenerative cases were profoundly assessed.

### Assessment of radiographs

All patients underwent MRI on a 1.5 T MR scanner (MAGNETOM Aera, Siemens AG, Erlangen, Germany) using the integrated spine coil. The T1-weighted sagittal, T2-weighted sagittal and axial images were acquired using a standard TSE sequence. Both T1 and T2 weighted lumbar MRI images from L1/2 to L5/S1 were profoundly analyzed in axial and sagittal planes.

**Fig. 1:**
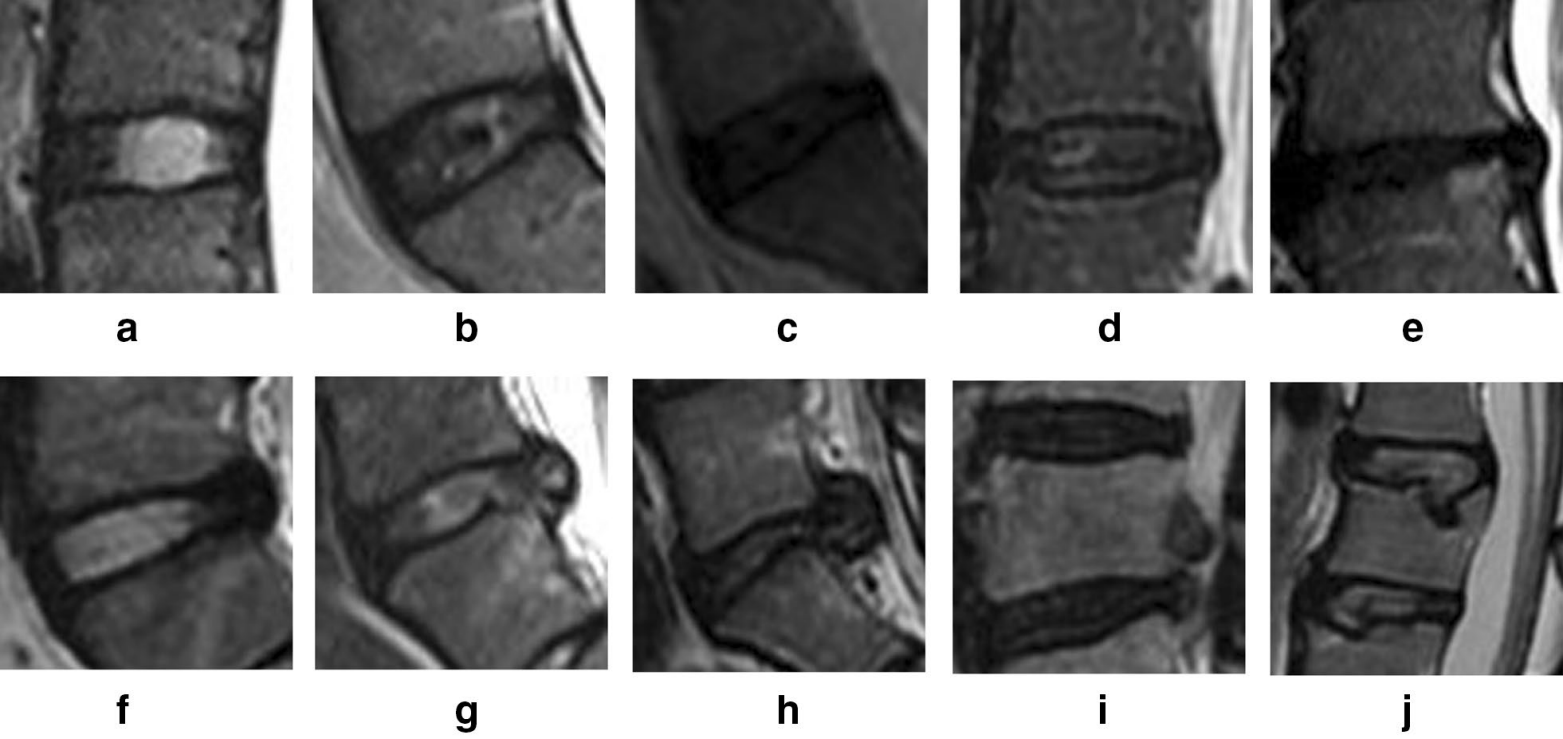
Mid-sagittal view on T2-weighted images of the Lumbardiscs. **a** (Normal), **b** (Blurred disc as early stage of LDD), **c** (Black disc), **d** (Black + bulging disc), **e** (Decreased height + protrusion disc), **f** (Pure bulging disc), **g** (Pure protrusion), **h** (Extrusion), **i** (sequestration), **j** (Schmorl node, SN)

**Fig. 2:**
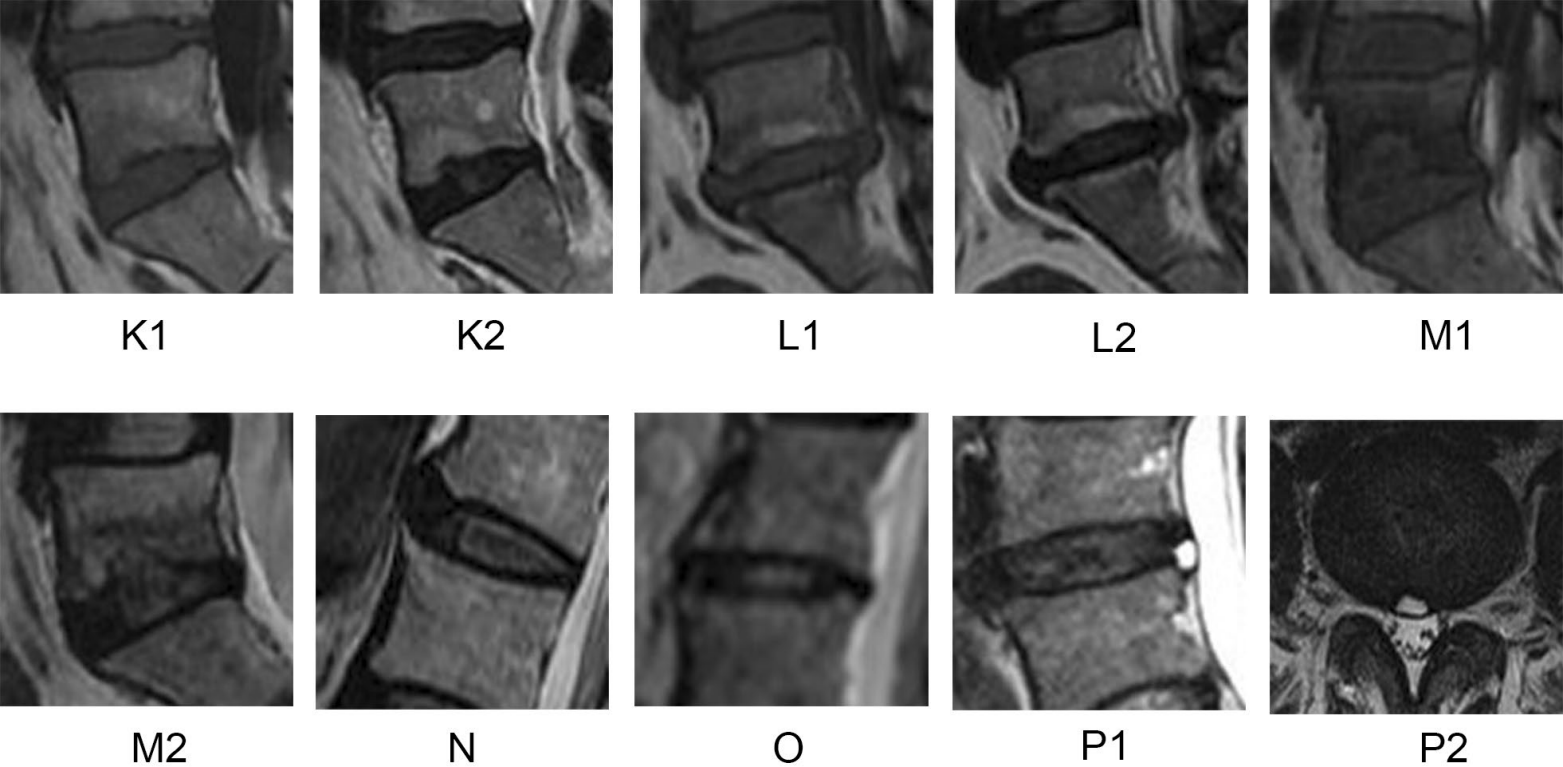
MRI of the Lumbardiscs.** K1** (Modic change type I on T1-weighted image),** K2** (Modic change type I on T2-weighted image),** L1** (Modic change type II on T1-weighted image),** L2** (Modic change type II on T2-weighted image),** M1** (Modic change type III on T1-weighted image),**M2** (Modic change type III on T2-weighted image),** N** (Spondylosis deformans),** O** (Pure decreased disc height),** P1** (Mid-sagittal viewof High intensity zone, HIZ), **P2** (Axial viewof High intensity zone, HIZ)

We classified lumbar discs in terms of the nomenclature version 2.0 (Fardon et al. 2014a, b) in combination of Pfirrmann grading (Pfirrmann et al. 2001) as follows: A (Normal), B (Blurred disc as early stage of LDD), C (Black disc), D (Black + bulging disc), E (Decreased height + protrusion disc), F (Pure bulging disc), G (Pure protrusion), H (Extrusion), I (sequestration), J (Schmorl node, SN), K (Modic change type I), L (Modic change type II), M (Modic change type III), N (Spondylosis deformans), O (Pure decreased disc height), P (High intensity zone, HIZ).Mid-sagittal view on both T1 and T2-weighted images of the Lumbardiscs’ phenotypes were shown in Figs. 1 and 2. Owing to the retrospective nature of the study, the assessed results could be compared with the radiographic reports signed by 2 radiologists. One experienced physician (JZ) specializingin spinal diseases assessed the radiographs. The same physician (JZ) re-assessed 100 randomly selected MRIs with over 1 month interval. Meanwhile, 100 another randomly selected MRIs were reviewed by another experienced physician (HQW).

We recorded the phenotypes using corresponding letters in a spreadsheet, besides the demographics of patients as age and gender.

According to the nomenclature of lumbar disc disease version 2.0, we define lumbar discs as Normal when all lumbar discs of a case were classified as A; LDD once one lumbar disc belongs to any type as B, C, J, K, L, M, N, O, or P; LDH if one lumbar disc is classified as F, G or H; LDD and LDH if one disc is D, E or I, or 1 disc as LDD with another as LDH in a case (Table 1).

**Table 1:** Prevalance of Phenotypes and classification of Lumbar discs

Phenotypes	Normal	LDD	LDH	LDD + LDH	Total (%)
A	√				
B		√		√	
C		√		√	
D				√	
E				√	
F			√	√	
G			√	√	
H			√	√	
I				√	
J		√		√	
K		√		√	
L		√		√	
M		√		√	
N		√		√	
O		√		√	
P		√		√	
Male (%)	704 (25.8)	415 (15.2)	163 (6.0)	1445 (53.0)	2727 (51.57)
Female (%)	608 (23.8)	411 (16.1)	115 (4.5)	1427 (55.7)	2561 (48.43)
Total (%)	1312 (24.8)	826 (15.6)	278 (5.3)	2872 (54.3)	5288 (100.0)
Age (SD)	24.7 (14.81)	49.76 (14.95)	37.14 (12.74)	51.31 (15)	
Average age	43.73 (18.69)				

### Statistical analysis

All data was recorded in a spreadsheet and analyzed with SPSS 19.0. Inter-class coefficient was used to assess inter-observer reliability. Reliability scores of <0.79, 0.80 to 0.89, and >0.90 were considered as poor, good, and excellent, respectively. Student *t* test was employed for measurement data; whereas enumeration data were analyzed using Chi square test. Lumbar disc degeneration and herniation in each segment were compared using binomial distribution test. A *P* value of <0.05 was considered statistically significant.

## Results

### Patients demographics

In total, the study included 5288 cases (26,440 lumbar discs) with lumbar MRI images, excluding 509 cases. The cross-sectional study of lumbar degeneration (COLD) consisted of 2727 males (51.57%) and 2561 females (48.43%). The average age of cases was 43.73 ± 18.69 years (range: 1–91 years, Table 1). The intra-observer and inter-observer reliability for lumbar disc phenotypes was 0.93 and 0.92, respectively.

### LDH and LDD epidemiology

The occurrence of LDHwas 14.18% in average (95% CI: 13.76%, 14.60%).For each segment, the occurrence of LDH from L1/2 to L5/S1 was 3.56, 6.22, 10.95, 26.08, and 24.09%, respectively. Moreover, the difference between each segment as well as with the average occurrence wasstatistically significant (*P* < 0.05). In herniated lumbar segments, L1/2, L2/3, L3/4, L4/5 and L5/S1 accounted for 5.01, 8.78, 15.44, 36.78, and 33.98% with statistical significance (*P* < 0.05).

**Fig. 3:**
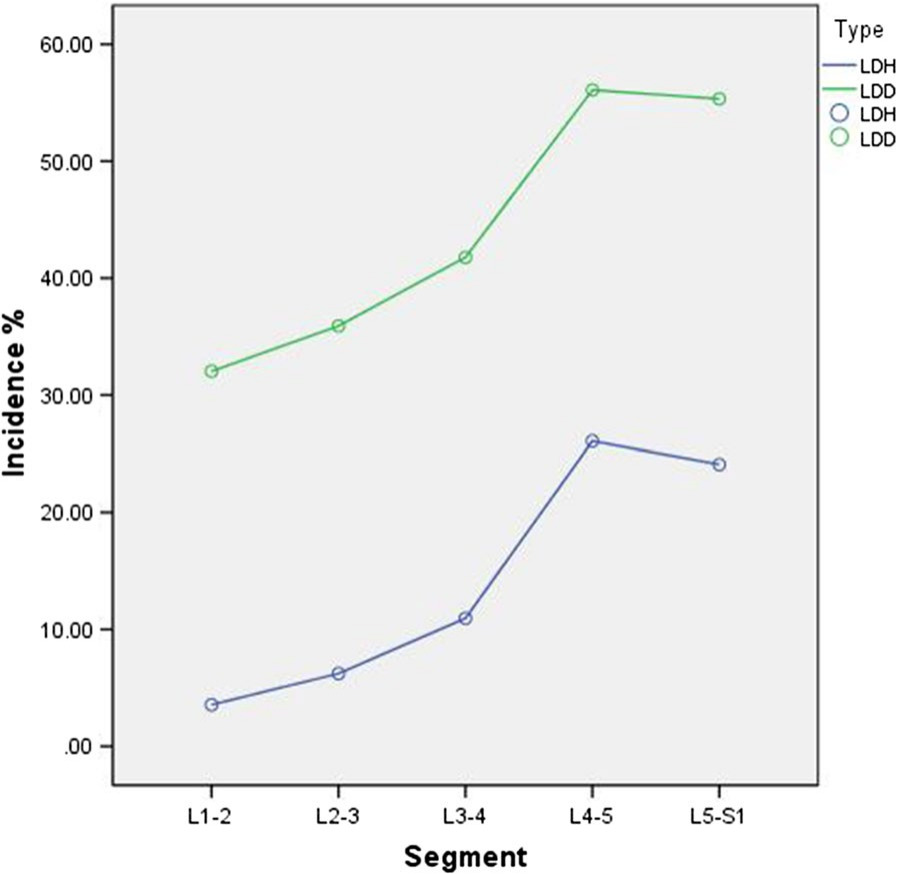
The comparison between LDH and LDD of occurrence incidence for each segment. From lumbar disc L1/2 to L5/S1,the incidence of LDH and LDD gradually increased, and the incidence rate of LDD is higher than LDH for each segment

**Fig. 4:**
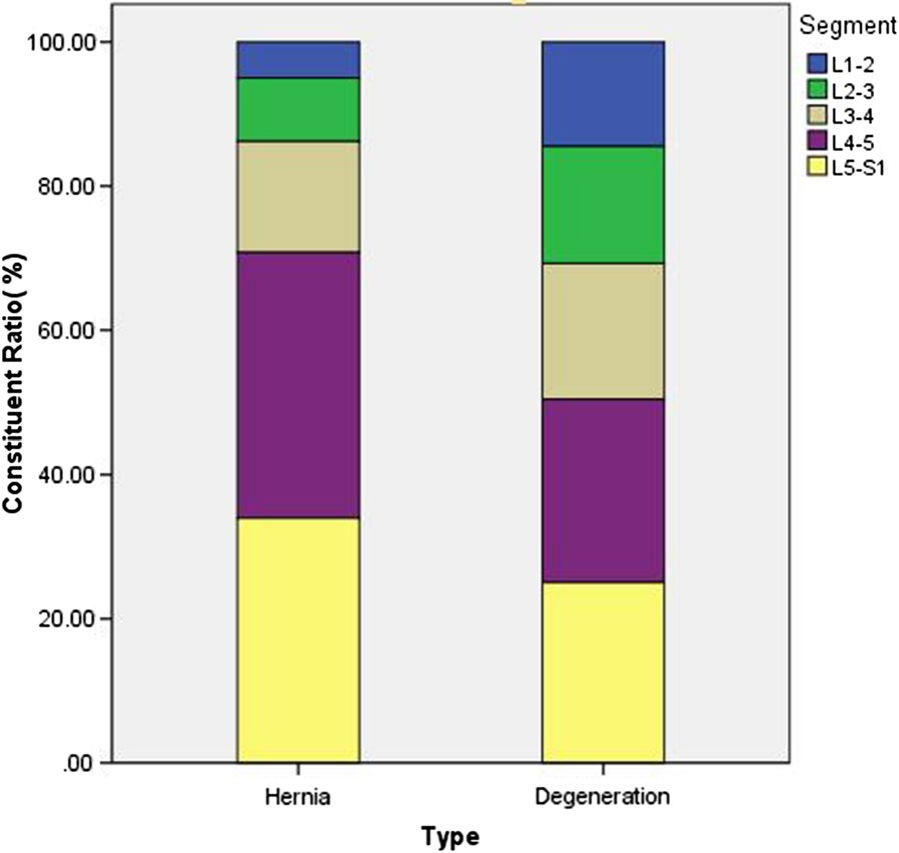
The comparison between LDH and LDD of constituent ratio for each segment. Both LDH and LDD, Lower lumbar disc (L4/5 and L5/S1) have higher proportion, especially of LDH

The occurrence of LDD was 44.23% in average (95% CI: 43.63, 44.83%). For each segment, the occurrence of LDD from L1/2 to L5/S1 was 32.03, 35.91, 41.77, 56.09, and 55.33%, respectively. Furthermore, the difference between each segment as well as with the average occurrence was statistically significant (*P* < 0.05). In degenerative lumbar segments, L1/2, L2/3, L3/4, L4/5 and L5/S1 accounted for 14.48, 16.24,18.89, 25.36, and 25.02% with statistical significance (*P* < 0.05).The occurrence and constituent ratio of LDD and LDH were shown in Figs. 3 and 4.

### Age impact

The mean age was 24.70 (±14.81) yrs for cases with normal lumbar discs; 49.76 (±14.95) yrs for cases with LDD; 37.01 (±12.91) yrs for LDH; 51.31(±15.00) years for LDD and LDH. The difference between each scenario was statistically significant (*P* < 0.05).

For each classification, the mean age of cases with normal discs was lower than those with abnormal discs; whereas cases with B, C, D, E, G, H, L, N, O and P types were older than those without these lesions (*P* < 0.05). The findings indicate that Modic changes, HIZ, spondylosis deformans and decreased disc height were linked with older age; whereas Schmorl node and lumbar disc sequestrationwere not associated with age (*P* < 0.05, Table 2).

**Table 2:** The mean age of positive and negative cases for each phenotype

Phenotypes	Positive +	Negative −	*t*	*P*
A	33.80 (16.14)	54.92 (14.59)	−111.08	0.000*
B	53.93 (12.51)	42.44 (18.94)	31.95	0.000*
C	58.17 (13.92)	41.11 (18.24)	56.51	0.000*
D	54.81 (14.34)	42.67 (18.71)	30.15	0.000*
E	56.59 (15.40)	42.69 (18.54)	32.26	0.000*
F	42.60 (13.72)	43.73 (18.76)	−1.29	0.200
G	48.29 (14.53)	43.51 (18.82)	8.48	0.000*
H	50.48 (15.34)	43.69 (18.70)	3.80	0.000*
I	44.75 (13.31)	43.71 (18.70)	0.51	0.614
J	44.93 (17.04)	43.70 (18.70)	1.00	0.316
K	46.64 (8.12)	43.71 (18.70)	0.50	0.604
L	51.26 (10.51)	43.70 (18.70)	2.36	0.018**
M	38.00 (−)	43.71 (18.69)	−0.31	0.760
N	71.56 (8.87)	43.68 (18.67)	8.96	0.000*
O	50.97 (16.76)	43.68 (18.69)	4.01	0.000*
P	48.81 (12.94)	43.69 (18.71)	3.10	0.002**

* *P* < 0.0001; ** *P* < 0.05

### Lumbar segment impact

Lumbar spine was classified into upper lumbar (L1/2, L2/3 and L3/4) and lower lumbar spine (L4/5 and L5/S1). In general, normal, blurred disc as early stage of LDD and Schmorlnodeoccurred more frequently in upper lumbar discs than lower lumbar discs. Moreover, other pathologic types occurred more frequently in lower lumbar discs than upper lumbar discs (*P* < 0.05, Table 3).

**Table 3:** Lumbar segment impact on each phenotype

Phenotypes	L1–L4 (%)	L4–S1 (%)	χ^2^	*P*
A	9873 (62.2)	4157 (39.3)	1339.9	0.000*
B	1905 (12.0)	1018 (9.6)	36.7	0.000*
C	2096 (13.2)	1937 (18.3)	127.7	0.000*
D	846 (5.3)	1433 (13.5)	543.8	0.000*
E	646 (4.1)	1307 (12.4)	636.8	0.000*
F	121 (0.8)	335 (3.2)	216.5	0.000*
G	295 (1.9)	852 (8.1)	587	0.000*
H	25 (0.2)	85 (0.8)	63.9	0.000*
I	9 (0.1)	74 (0.7)	83.8	0.000*
J	202 (1.3)	34 (0.3)	65	0.000*
K	1 (0.0)	10 (0.1)	11.9	0.001**
L	3 (0.0)	31 (0.3)	37.1	0.000*
M	0 (0.0)	1 (0.0)	0.221	0.400
N	23 (0.1)	13 (0.1)	0.227	0.384
O	71 (0.4)	35 (0.3)	2.162	0.141
P	29 (0.2)	99 (0.9)	74.7	0.000*
Total	16,145 (100)	11,421 (100)		

Pathologic types D–L and P occurred more frequently in lower lumbar discs than upper lumbar discs; Whereas A–C and J (normal, blurred disc as early stage of LDD and Schmorl node) occurred more frequently in upper lumbar discs

* *P* < 0.0001;** *P* < 0.05

## Discussion

The study is the first providing novel vision on the prevalence and landscape of LDD and LDH in lumbar spine based the largest COLD samples, shedding valuable lights on lumbar disc diseases. Moreover, the mega-data of lumbar spine images are on the base of the most updated version of lumbar disc nomenclature released by the authorized society (Fardon et al. 2014a, b).

Indeed, LDD and LDH are amongst the most common form of spinal diseases, tightly linked with low back pain. Despite the widespread impact on people’s daily lives, the accurate prevalence and underlying relations with age remain elusive. In summary, the prevalence of lumbar disc degeneration is 44.23%, higher than LDH as 14.18%. L4/5 and L5/S1 are the most frequent involved segments for the majority of lumbar disc diseases. SN occurs (1.6%) more frequently in upper lumbar spine, independent of age. Modic changes (0.87%) are closely related with older age. Moreover, Modic II is the most common type.

### Prevalence of LDD and other phenotypes

In 2014, Teraguchi et al. reported the prevalence of LDD is highest in L4/5 as 69.1% for men and 75.8% for women (Teraguchi et al. 2014). Their study was based on the Wakayama Spine Study with 975 participants. In 2015, the same research team reported that LDD prevalence as 30.4%, SN as 1.5% in terms of the same cross-sectional study (Teraguchi et al. 2015). Samartzis et al. noted LDD prevalence as 72.7% based on 2599 southern Chinese volunteers (mean age 41.9 years). It should be stressed that Teraguchi et al. determined LDD grading in terms of Pfirrmann’s 5-grade scheme on T2-weighted images (Pfirrmann et al. 2001) (grading system on 300 lumbar discs); whereas Samartzis et al. judged LDD based on 4-grade system proposed by Schneiderman et al. (1987) (grading system on 180 lumbar discs).

We reported LDD and LDH separately based on the updated version, considering the aforementioned grading schemes. The prevalence varies between our cross-sectional study and the Wakayama Spine Study, southern Chinese subjects, due to several factors. First, the grading criteria are different. Notably, there is significant variability in the interrater and intrarater agreements of MRI in assessing degenerative conditions of the lumbar spine even with standardized evaluation criteria (Fu et al. 2014). In particular, as we pointed out (Li et al. 2015), phenotypes, including SN, Modic changes, HIZ, have been relegated into LDD according to the updated version. Therefore, studies using previous MRI grading schemes might not accurately reflect the state-of-the-art concept of lumbar disc disease. On the other hand, it should be stressed that definition of a normal lumbar disc is a relative notion (Li et al. 2015). In general, current relative signal intensity in MRI is the gold standard for classifying LDD, upon which we diagnose the lumbar spine as normal or LDD. Strikingly, lumbar discs are among the early degenerative organs in the body, even in the first decade (Roberts et al. 2006). The degeneration sign initiates from cell phenotype conversion from notochord cells to small chondrocyte-like nucleus pulposus cells localized within nests (Chen et al. 2013). If we judge lumbar spine discs in terms of more sensitive molecular or RNA expression profiling criteria as we addressed previously (Sun et al. 2013; Wan et al. 2014; Wang et al. 2010, 2011), even early degeneration as phenotype B would be diagnosed as LDD. However, grading 1 to grading 3 would generally be considered as Normal according to MRI grading schemes (Pfirrmann et al. 2001; Schneiderman et al. 1987). Therefore, the grading system of LDD should be integrated for the scientific community.

Second, the sample size should not be overlooked. Apparently, largersample size results in aconclusion with higher confidential level; despite large sample exploration is time-consuming. At this point, the prevalence based on 26,440 lumbar discs with a wide age range might be more reliable.

### LDD and LDH

Despite LDD and LDH are highly linked with the same OMIM code (Song et al. 2013), they are not exactly the same disease (Wang and Samartzis 2014). LDD is characterized by progressive loss of aggrecan (Le Maitre et al. 2009), annular fibrosis rupture (Kazezian et al. 2015; Pirvu et al. 2015), collagen type transformation, cartilage endplate alterations (Arpinar et al. 2015) and decreased disc height (Jarman et al. 2015). In MRI, typical LDD represents as black discs. However, LDH can occur in adolescents without typical signs of LDD (Lagerback et al. 2015). The prevalence of LDH-related sciatica has been reported as 2% in adults (Younes et al. 2006). However, cases with LDH as bulging or mild extrusion, protrusion might not have clinical sciatica. At this point, the study presents the first line of evidence, unraveling the prevalence of LDH as 14.18%. Moreover, LDH prevalence for each segment has been clarified.

### SN hallmarks

In our study, SN occurs in 1.6% of all cases, consistent with the results of the Wakayama Spine Study (Teraguchi et al. 2015). Previously, it remains unclear whether SN occurs in upper or lower lumbar spine more frequently. We noted that SN occurs more frequently in upper lumbar spine than lower lumbar spine, providing novel insights in SN. The underlying mechanisms might be partly due to the anatomic hallmarks of posterior longitudinal ligament as main reinforcement ending in L3 (Wang and Samartzis 2014).

### Modic changes

In 1988, Modic and colleagues defined changes in vertebral body marrow via MRI imaging. The prevalence of type I changes was 4% among 474 patients; whereas the prevalence of type II changes was 16% (Modic et al. 1988). Thereafter, a number of studies addressed the issue with differences, ranging from 0 to 22% in normal population, 6.3–60% in patients (Jensen et al. 2008; Maatta et al. 2015). In the current study, the prevalence of Modic changes was lower in comparison with the aforementioned studies. The heterogeneousness in sample size and objects of studies contributes to the variety of reports. Therefore, the definite prevalence of Modic changes needs well defined.

Despite our study shed novel light on the understandings of lumbar disc disease, we acknowledge that several limitations exist. The retrospective image study nature, as well as institutionalbased subjects might weaken the strength of the study. Further research with normal volunteers might better clarify the definite feature of lumbar disc disease.
